# Monthly Reports

**Published:** 1803-10-01

**Authors:** J. Ricards

**Affiliations:** Jermyn Street, St. James's


### MONTHLY REPORTS.

*Cases admitted under the Care of the Surgeon of the Finsbury Dispensary, St. John's Square, Clerkenwell, from August 10, to September 10, 1803.*

| | | | |
|---|---|---|---|
| Phlegmone - - - - - | 1 | Fractura Ulnæ - - - - | 1 |
| Abscessus Artuum - - | 3 | Vulnus - - - - - - - | 1 |
| ——— Maxilla - - | 1 | Luxatio Humeri - - - | 1 |
| Mastodynia - - - - - | 4 | Lues - - - - - - - - | 3 |
| Hernia Humoralis - - | 2 | Gonorrhœa - - - - - | 2 |
| Ulcera Cruris - - - - - | 11 | Scrophula - - - - - - | 3 |
| Leucoma - - - - - - | 1 | Ascites - - - - - - - | 2 |
| Contusiones - - - - | 9 | Hydarthus Genu - - - | 2 |
| | | Sarcocele | |

---

| | | | |
|---|---|---|---|
| Sarcocele - - - - - - | 2 | Psora - - - - - - - - | 1 |
| Hernia - - - - - - - - | 3 | Eruptio Chronica - - | 1 |
| Prolapsus Ani - - - - | 1 | | — |
| Arthrodynia - - - - - | 3 | Total - | 60 |
| Tinea - - - - - - - - | 3 | | — |

A few months since, some observations were made by the Reporter respecting applications of a cold nature to local inflammations of a specific kind, as Gout and Rheumatism, which are generally considered as diseases whose existence should be rather encouraged than repelled, and which are thus very commonly protracted to a very tedious length.

We are much indebted to a physician of great discrimination and ability (Dr. Kinglake) for the remarks and relation of facts on this subject, which have recently been presented to the public by the medium of the Medical and Physical Journal, and whose assiduity in the collection of well substantiated cases, in which similar treatment has been pursued with success, will in all probability add much to our knowledge with respect to the nature of these diseases, and to our capability in the removal or mitigation of them.

Several cases have come within my knowledge, since those which have already been alluded to, in which the frigorific process has been attended with manifest advantage in recent cases of Gout and Rheumatism, or in such cases as are evidently accompanied with vigorous action, and inflammation of the sthenic character; in no one of the cases in which this treatment has been pursued, has any ill consequences succeeded to my knowledge; yet these certainly cannot be considered as sufficient evidence to prove its validity in all cases, but sufficient to warrant a continuance of the experiment in all cases of the above description.

A surgeon of considerable practice in this metropolis, a man of real professional ability, who has been a martyr to the pain and confinement of the Gout, is continually in the habit of arresting its progress when he has an opportunity of performing it sufficiently early, by a practice of this kind. When he perceives the approach of his enemy, he goes to the Thames, and making bare his legs and feet, immerses them in the river from the side of a boat, in which he is rowed about for a considerable time, which seldom fails of producing the effect required.

Several cases which have come to my knowledge, in which the feet have in the commencement of the attack of Gout

Gout been accidentally exposed to cold and moisture, with with great advantage, further tend to justify the belief that such treatment will in a great variety of cases prove beneficial; though from the contrary nature of its operation to the plan which has for the most part been hitherto adopted, it is probable that much time must elapse before the remedy can become general.

*Jermyn Street, St. James's.* J. RICARDS.

